# Nucleofugal behavior of a β-shielded α-cyanovinyl carbanion

**DOI:** 10.3762/bjoc.14.281

**Published:** 2018-12-11

**Authors:** Rudolf Knorr, Barbara Schmidt

**Affiliations:** 1 Department Chemie, Ludwig-Maximilians-Universität München, Butenandtstrasse 5–13 (Haus F), 81377 München, Germany

**Keywords:** alkoxide fission, desilylation, fragmentation, retro-addition, reversible carbonyl addition, shielded acrylonitrile

## Abstract

Sterically well-shielded against unsolicited Michael addition and polymerization reactions, α-metalated α-(1,1,3,3-tetramethylindan-2-ylidene)acetonitriles added reversibly to three small aldehydes and two bulky ketones at room temperature. Experimental conditions were determined for transfer of the nucleofugal title carbanion unit between different carbonyl compounds. These readily occurring retro-additions via C–C(O) bond fission may also be used to generate different metal derivatives of the nucleofugal anions as equilibrium components. Fluoride-catalyzed, metal-free desilylation admitted carbonyl addition but blocked the retro-addition.

## Introduction

**Scheme 1:**
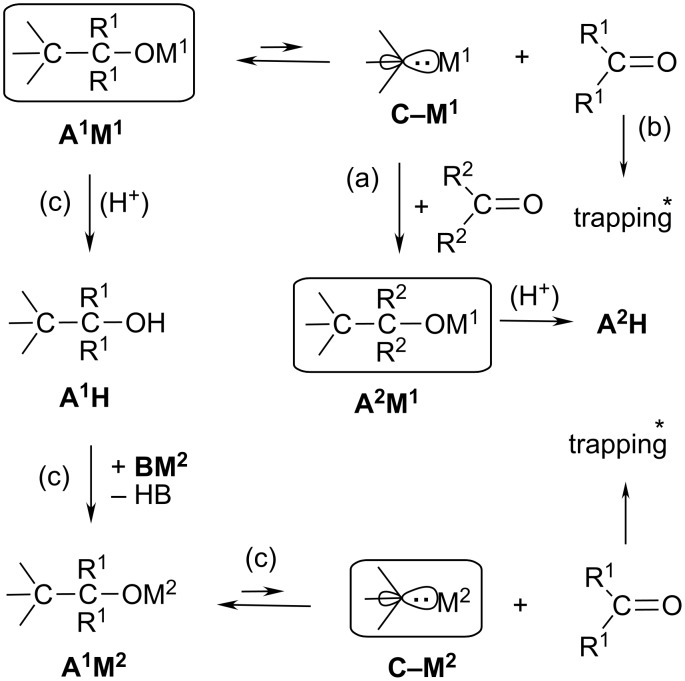
The nucleofugal carbanion unit **C** escapes from the alkoxides **A^1^M^1^** or **A^1^M^2^** (M = metal), generating the organometallics **C–M^1^** or **C–M^2^**, respectively. Route (a): **C–M^1^** can be trapped by (R^2^)_2_C=O to form the new alkoxide **A^2^M^1^**. Route (b): Trapping (*) of (R^1^)_2_C=O might accumulate **C–M^1^** or **C–M^2^**. Route (c): A basic reagent BM^2^ replaces M^1^ by M^2^ and may generate **C–M^2^**. Achiral alkoxides (**A**) are depicted for simplicity.

A carbanionic fragment shows nucleofugal behavior on breaking its single-bond connection with a developing electrophilic center. Apart from deprotonation reactions or formation of a separated ion pair and other trivial examples, the (at least formally) heterolytic cleavage of C–C single bonds can provide cases of interest if it generates organometallic compounds under unusual conditions. The well-known cases of alkoxide fission [1–3] (top line of Scheme 1) may be viewed as a reversed formation of an alkoxide **A^1^M^1^** from an organometallic **C–M^1^** and a carbonyl compound (R^1^)_2_C=O. Such a C–C fission (retro-addition reaction) can occur already near room temperature (rt) if the nucleofugal carbanion proves to be an electronically stabilized N≡C–CH_2_**^–^** [1] or allylic [2] species or a short-lived equilibrium component [3].

A cleavable alkoxide **A^1^M^1^** can be employed in at least three ways: (a) its equilibrium component **C–M^1^** may be trapped by a different carbonyl compound (R^2^)_2_C=O to give the new alkoxide **A^2^M^1^** (and from there the alcohol **A^2^H**) as evidence for the intermediacy of a short-lived species **C–M^1^**. If **A^2^M^1^** is also cleavable, the two alkoxides **A^1^M^1^** and **A^2^M^1^** may be obtained under thermodynamic control. (b) Trapping of the other equilibrium component (R^1^)_2_C=O by a nucleophile might accumulate the organometallic compound **C–M^1^**. (c) **A^1^M^1^** may be used to replace **M^1^** by **M^2^** with intent to study a different organometallic **C–M^2^** (bottom part of Scheme 1). For this purpose, **A^1^M^1^** should be demetalated through work-up to give, for instance, the alcohol **A^1^H**; the subsequent deprotonation of **A^1^H** by an **M^2^**-containing base **BM^2^** must be complete before the generated alkoxide **A^1^M^2^** begins to release **C–M^2^** that would otherwise be protonated by any unconsumed portion of **A^1^H**. We had used this technique [3] to generate the short-lived, NMR-invisible pivaloylmetals (H_3_C)_3_C–COM from the overburdened alkoxides of tri-*tert*-butylacyloin that were readily cleaved with relief of internal strain as a driving force. We now report here on the above-mentioned options (a) and (c) with the focus on some nucleofugal traits of the carbanion unit of the stable [4], β-shielded α-metalated acrylonitriles.

## Results and Discussion

**Scheme 2:**
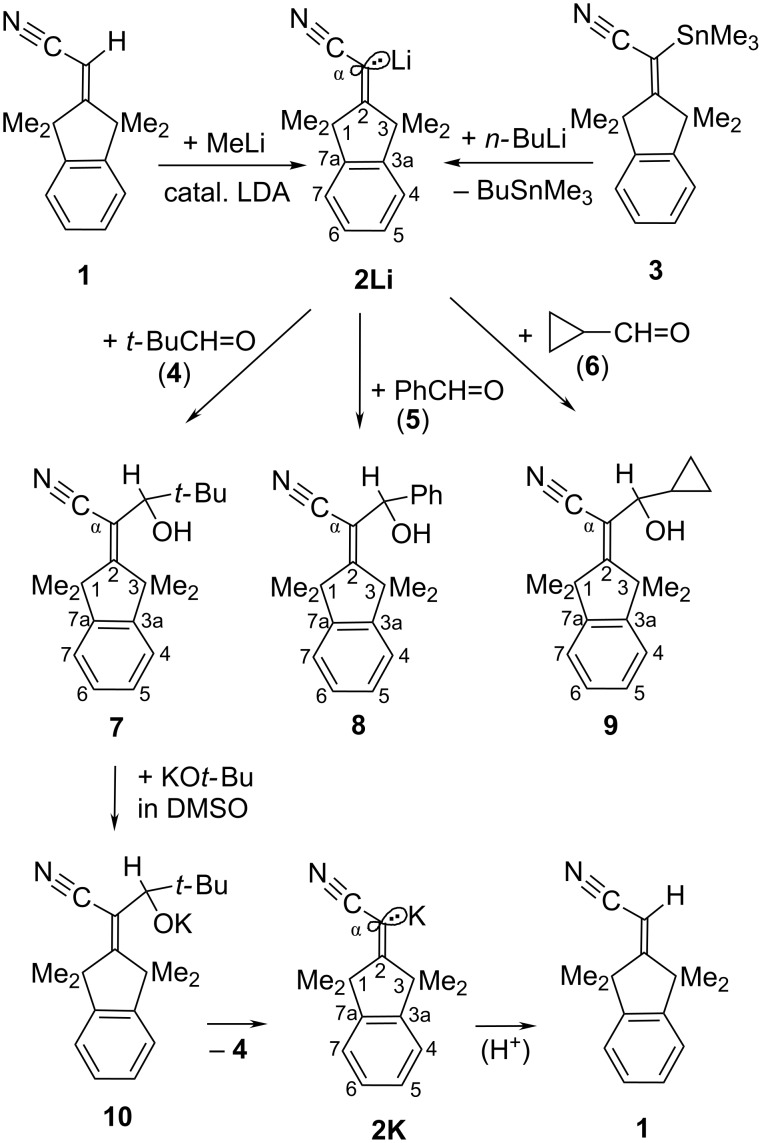
Preparation of β-shielded α-lithioacrylonitrile **2Li** and its reaction with aldehydes **4**–**6** to adducts **7**–**9**. Cleavage of the potassium alkoxide **10** (bottom line) gave **2K** and then **1**.

The α-cyanoalkenyllithium **2Li** (top line of Scheme 2) may be prepared [4] either by LDA-mediated deprotonation (LDA = iPr_2_NLi) of the β-shielded acrylonitrile derivative **1** or by an Sn/Li transmetalation reaction of *n*-butyllithium (“*n*-BuLi”) with the α-stannyl derivative **3**. Due to intermolecular LiN coordination, **2Li** was deduced [4] to form a clustered ground state that showed no obvious rate anomaly with previously [4] studied electrophiles; this should also hold true here for pivalaldehyde *t*-BuCH=O (**4**, “*t*-Bu” = *tert*-butyl). The chiral adduct **7** of **4** exhibited four different NMR signals for the four methyl groups at the 1- and 3-positions. Analytically pure **7** was obtained only through distillation as a crystalline sample that decayed during an attempted recrystallization from methanol. A possible reason for this instability at a temperature far below the boiling point became clear on addition of some solid potassium *tert*-butoxide (KO*t*-Bu) to an NMR tube that contained purified **7** in DMSO at rt: After the immediate appearance of a yellow tint, the next ^1^H NMR spectrum revealed that **7** was almost completely transformed into the acrylonitrile derivative **1** (bottom line of Scheme 2). This suggested that the nucleofugal carbanion unit of **2K** had escaped from the potassium alkoxide **10** with formation of **2K** (yellow tint) and *t*-BuCH=O, whereupon **2K** was trapped by proton sources such as DMSO or unconsumed **7** to produce **1**. As a consequence, the general procedure (GP) of adding carbonyl compounds to deprotonated **1** commends to use an acidic work-up and a strict exclusion of bases that are stronger than diluted aqueous NaHCO_3_.

Benzaldehyde (**5**) and cyclopropanecarboxaldehyde (**6**) added also readily to **2Li** with production of **8** and **9**, respectively. As already reported above for **7**, adducts **8** and **9** showed also four CH_3_ NMR signals on account of the stereogenic carbon centers C–OH. The NMR AB-type interproton ^3^*J* splittings of the H–C–O–H moieties in **8** and **9** disclosed that intermolecular scrambling of the hydroxy protons was retarded through steric congestion.

**Scheme 3:**
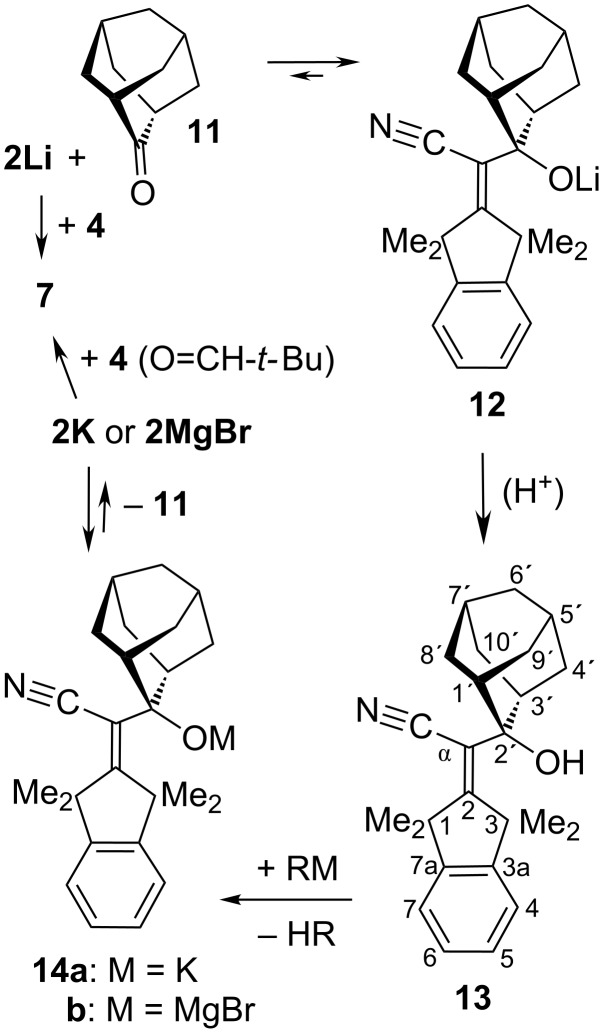
Reversible formation of the adduct **13** of adamantan-2-one (**11**).

The adduct **13** (Scheme 3) of adamantan-2-one (**11**) was formed (like the above aldehyde adducts) through addition at C-α (rather than at nitrogen), as shown by the δ values of the tetramethylindane part which were consistent with those of **7**–**9**. Complete NMR assignments for the 2-hydroxyadamantan-2-yl part of **13** were achieved with the two-dimensional NOESY and HSQC techniques at rt. Except for the OH signal, all other resonances were changed only insignificantly on cooling to −60 °C. Cleavage of **13** via the lithium alkoxide **12** (top line of Scheme 3) was fast on the laboratory time scale at rt in the solvents THF, Et_2_O, *t*-BuOMe, or toluene. As mentioned in the Introduction, the successful transfer of **2Li** from **11** to **4** depends on the exclusion of proton sources (including the alcohol **13**) that would protonate **2Li** with formation of **1**. Thus, the slow addition of **13** to a well-stirred solution of methyllithium (MeLi, 2 equiv) liberated gaseous CH_4_ (1 equiv) so that **13** was completely consumed before the electrophile *t*-BuCH=O (**4**; 4 equiv) was introduced and furnished adduct **7** but no trace of **1**. The worst case (with **1** as a preponderant product) may be encountered when the deprotonating base is added to the alcohol **13** either too slowly or in a less than stoichiometric amount. For instance (bottom line of Scheme 3), the heterogeneous, slow deprotonation of **13** in THF by the insoluble base potassium hydride (KH) afforded **11** and **1** as the only products via cleavage of the potassium alkoxide **14a** and protonation of the emerging **2K** by residual **13**, so that the final introduction of pivalaldehyde (**4**) produced no adduct **7**. However, the same result was also found when **13** was added to a homogeneous solution of PhCH_2_K in THF; this suggested that **13** had transferred its proton to the emerging, thermally stable [4] cleavage product **2K** faster than to the residual PhCH_2_K (perhaps a problem of slow mixing). On the other hand, deprotonation of **13** by the Grignard reagent MeMgBr in THF was complete (quantitative CH_4_ evolution) within 20 min at 0 °C; the subsequent cleavage reaction of the generated magnesium alkoxide **14b** was very slow at rt, creating **2MgBr** (Scheme 3) in the presence of *t*-BuCH=O (**4**) and therefrom **7** together with only a small amount of **1**. This almost clean production of **7** agrees with the versatile [5] reactions of H_2_C=C(CN)–MgBr with many electrophiles. In most of the above trapping experiments, a slow disproportionation of *t*-BuCH=O was observed to generate *t*-BuCH_2_OH and *t*-BuCO_2_Li as side-products.

**Scheme 4:**
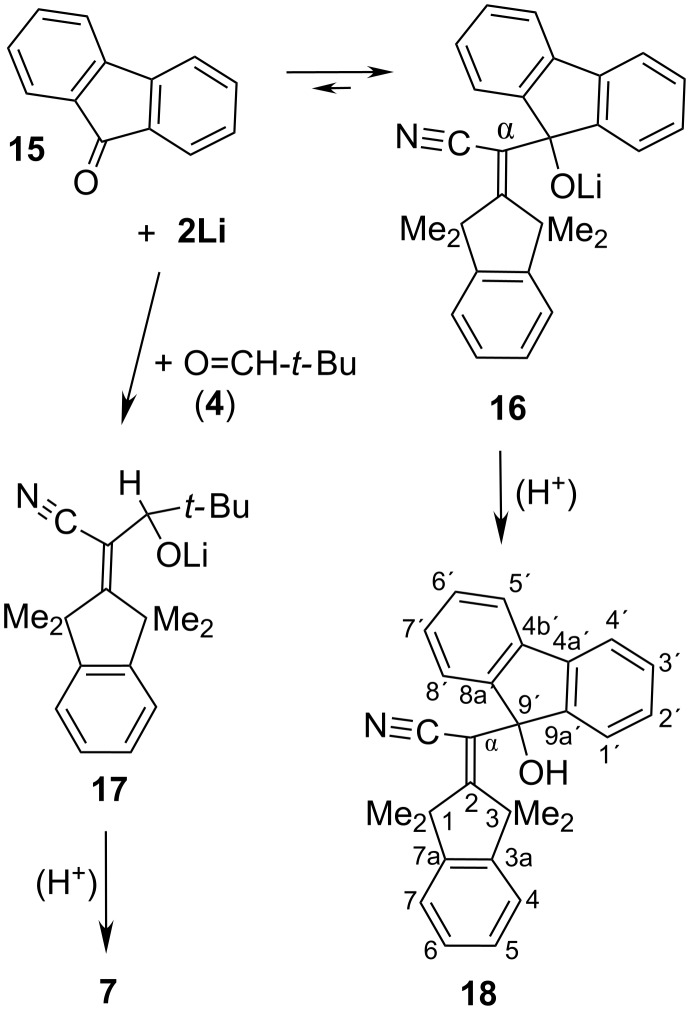
Preparation and cleavage of the adduct **18** of fluoren-9-one (**15**).

The adduct **18** (Scheme 4) of fluoren-9-one (**15**) had again a triangular ligand-binding system at C-α, as shown by the completely assigned NMR resonances. As the only unexpected contrast to **7**–**9** and **13**, the 3-CH_3_ protons were observed to absorb at a higher δ value (1.98 ppm) than that of the 1-CH_3_ protons, which amounts to an increase of 0.34 ppm relative to 3-CH_3_ of **13**. We ascribed this to a ring-current effect by the 9´-hydroxyfluoren-9´-yl substituent that can be expected to prefer a perpendicular conformation relative to the indane rings with the 9´-OH group pointing toward 3-CH_3_. As a peculiar line-width effect, the NMR signals of 3-CH_3_ (^1^H and ^13^C) and of 4-H were significantly broadened at rt but had the usual narrow line widths at −45 °C. Formation of the primary lithium alkoxide **16** (Scheme 4) was shown to be readily reversible as follows. Without protolysis and work-up of the green-colored solution, **16** was kept in THF at rt for 30 min and then treated with *t*-BuCH=O (**4**; 1.5 equiv); after a further period of 2 hours at rt, the GP work-up furnished the adduct **7** of *t*-BuCH=O along with the co-product fluoren-9-one (**15**), some fluoren-9-ol [6], and a small amount of **1**, but no **18** (would derive from residual **16**). Thus, **16** had released the nucleofugal carbanion unit of **2Li** which was trapped by **4** to give the alkoxide **17** of **7**.

**Scheme 5:**
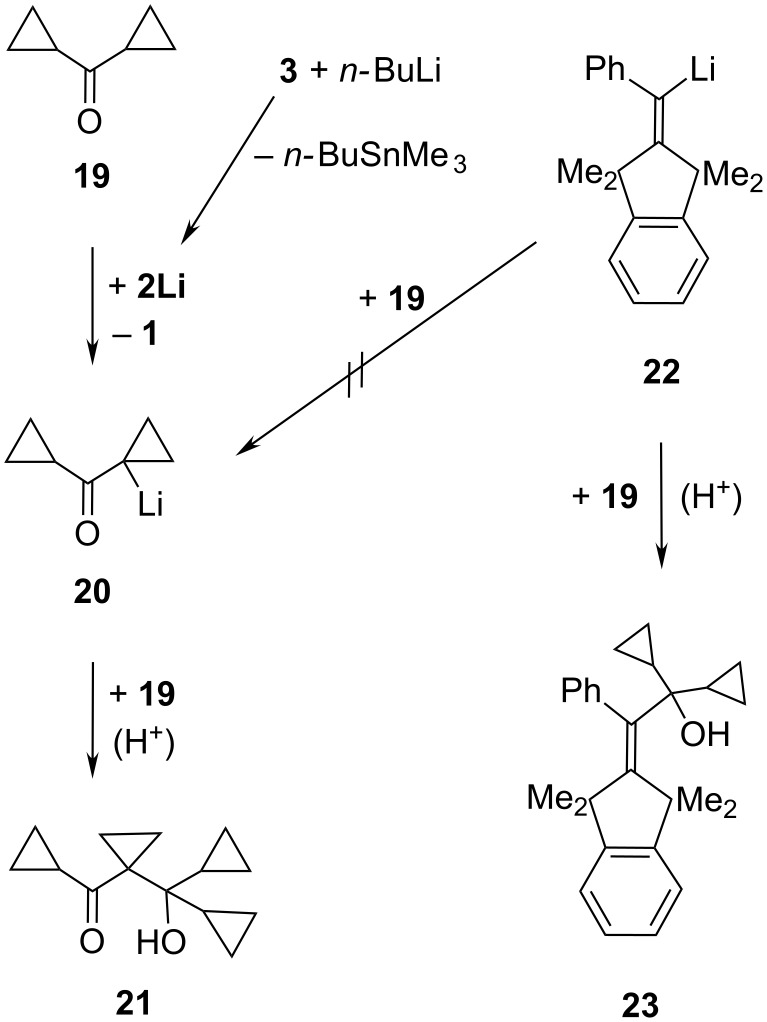
Proton transfer from dicyclopropyl ketone (**19**) to **2Li**.

In view of the apparently modest spatial demand of the α-cyano substituent in the adducts of **2Li**, it seemed surprising that the following ketones generated no (or no kinetically stable) C=O adducts at rt: *t*-Bu_2_C=O, pivalophenone (*t*-Bu–C(O)–Ph), benzophenone (Ph_2_C=O) [7], bis(1-methylcyclopropyl)ketone [8], and dicyclopropyl ketone (**19**). Instead of an adduct of **19** to **2Li**, the carboxyalcohol **21** (a known [9] product of **19** with LDA) in Scheme 5 and the acrylonitrile derivative **1** were obtained from **19**; this run was conducted in the absence of LDA with a sample of **2Li** that had been prepared in Et_2_O through an Sn/Li interchange reaction [4] of **3** with *n*-BuLi. Therefore, **2Li** should have deprotonated **19** with formation of **20** that was trapped by **19** to give **21**. But why was **21** not formed from **19** with the α-phenylalkenyllithium **22** (related to **2Li**) that produced [10] the “normal” carbonyl adduct **23** of **19**? This “normal” C=O addition reaction was obviously faster than the deprotonation of **19** and also apparently irreversible under the reaction conditions, whereas the unobserved C=O addition of **19** to **2Li** might be possible yet quickly reversible with a terminating proton transfer from **19** to **2Li** as shown in Scheme 5. Alternatively, carbonyl additions to **2Li** might be generally retarded (relative to protonations) on account of the clustered [4] ground state structure of **2Li** in solution.

**Scheme 6:**
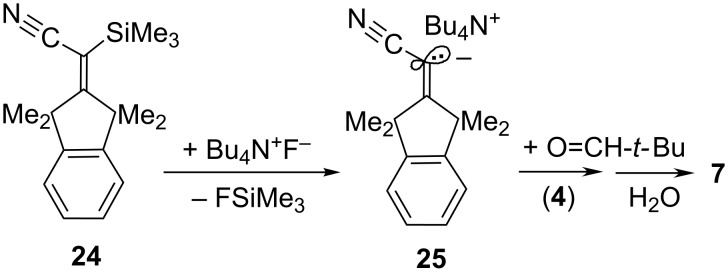
Metal-free release of the carbanion unit in **25** and its seizure by *t*-BuCH=O (→ **7**); Bu = *n*-butyl.

Electrophilic cations (**Li^+^** or **K^+^**) are not necessary (albeit perhaps helpful) for the addition of the carbanion unit of **2Li** or **2K** to carbonyl compounds: Generated through desilylation of **24** (Scheme 6) by tetrabutylammonium fluoride (Bu_4_N^+^F^−^; ≤0.05 equiv), the ion pair **25** was trapped by pivalaldehyde (**4**) with formation of **7** along with a comparable amount of the alkene **1**. However, the primary alkoxide product, as formed by **25** and **4**, was supposedly blocked by FSiMe_3_ and hence unsuitable for an analysis of the retro-addition reaction (see Supporting Information File 1).

## Conclusion

(i) The nucleofugal carbanion (α-deprotonated **1**) can escape with surprising ease from alkoxides, perhaps with some assistance by a metal cation if present. Conversely, metal cation assistance was not necessary for the rapid carbanion release from the α-silyl compound **24** in the presence of Bu_4_N^+^F^−^ in catalytic amounts.

(ii) Most of the above alkoxide fission reactions were conducted in the presence of an electrophile for trapping the released nucleofugal carbanion; this provided a first evidence for the retro-addition process with an alkenylmetal intermediate. These fissions were slow in case of the magnesium alkoxide but rapid for the lithium or potassium alkoxides at ambient temperatures.

(iii) The alternative trapping [3] of the carbonyl component by means of a nucleophile was not tried here but might serve to accumulate the organometallic equilibrium component for the purpose of spectroscopic characterization or X-ray diffraction analyses.

(iv) Fission of the lithium alkoxide of the adduct **13** of adamantan-2-one appeared to be comparably fast on the laboratory time scale in the solvents THF, Et_2_O, *t*-BuOMe, or toluene. A similarly weak solvent dependence had been observed [4] for the heterolytic *cis*/*trans* stereoinversion of **2Li**.

(v) For more profound mechanistic investigations, one should be aware of the established [4] clustered ground state of **2Li** and the possibility that clustered species might be the reactive components even in THF solutions, whereas the retro-addition processes may perhaps involve monomeric nucleofuges.

## Experimental

**General remarks.** Stoichiometric formation of alkoxides through deprotonation of the alcohols was conveniently performed with methyllithium or methylmagnesium bromide and gas-volumetric control of the liberated methane (ca. 25 mL/mmol at 22 °C). All ^1^H and ^13^C NMR shifts δ were referenced with internal Me_4_Si. NMR abbreviations were as follows: d = doublet, m = multiplet, q = quartet, quat = quaternary, s = singlet, t = triplet.

**General procedure (GP) for carbonyl addition to α-lithio-α-(1,1,3,3-tetramethylindan-2-ylidene)acetonitrile (2Li).** A dry Schlenk flask was charged with a magnetic stirring bar, anhydrous Et_2_O or toluene (2 mL), and *N*,*N*-diisopropylamine (0.161 mL, 1.14 mmol). This solution was stirred at –30 °C under argon gas cover during the addition of *n*-BuLi (1.05 mmol) in hexane (0.47 mL). The created LDA (up to 1.05 mmol) solution was treated with the solid nitrile **1** (200 mg, 0.95 mmol) and stirred further at rt till **1** was completely dissolved, whereupon the carbonyl compound (1.24 mmol, neat or in a solvent) was added to the yellow solution of **2Li** and stirred for another 30 min. The mixture was poured into aqueous HCl (2 M, 10 mmol) and extracted with Et_2_O (3 × 20 mL). (Note that the product might decay in a non-acidic milieu.) The combined Et_2_O extracts were shaken first with water (1 × 20 mL), then with diluted aqueous NaHCO_3_, and again distilled water (2 × 20 mL), then dried over Na_2_SO_4_, filtered, and concentrated.

**3´-Hydroxy-4´,4´-dimethyl-2´-(1,1,3,3-tetramethylindan-2-ylidene)pentanenitrile (7).** The GP protocol was used to treat LDA (1.56 mmol) in toluene with the β-shielded acrylonitrile **1** (300 mg, 1.42 mmol), followed by pivalaldehyde (**4**, 0.200 mL, 1.84 mmol). After work-up, the crude material (401 mg) distilled at 120–130 °C (bath temp.)/0.03 mbar to give pure **7** (132 mg, 29%) with mp 108–112 °C; **7** would decay on recrystallization. ^1^H NMR (CDCl_3_, 400 MHz) δ 1.16 (s, 9H, *t*-Bu), 1.59 and 1.61 (2 s, 2 × 3H, 2 × 3-CH_3_), 1.69 and 1.71 (2 s, 2 × 3H, 2 × 1-CH_3_), 1.97 (broad s, 1H, OH), 4.71 (s, 1H, H–CO), 7.14 and 7.17 (2 m, 2 × 1H, 4-/7-H), 7.27 (m, 2H, 5-/6-H) ppm, assigned through comparison with **8**; ^13^C NMR (CDCl_3_, 100.6 MHz) δ 27.0 (q, *Me*_3_C), 28.4 and 28.7 (2 q, 2 × 1-CH_3_), 30.9 and 32.4 (2 q, 2 × 3-CH_3_), 35.7 (quat, Me_3_*C*), 49.42 (quat, C-3), 49.57 (quat, C-1), 74.6 (d, HCO), 110.9 (quat, C-α), 118.3 (quat, C≡N), 122.1 (d, C-4), 122.4 (d, C-7), 127.7 and 127.9 (2 d, C-5/-6), 147.6 (quat, C-7a), 148.0 (quat, C-3a), 180.6 ppm (quat, C-2); IR (KBr): 3475 (O–H), 2927, 2869, 2214 (s, C≡N), 1610, 1487, 1464, 1368, 1274, 1192, 1060, 1013, 762 cm^−1^; anal. calcd for C_20_H_27_NO (297.4): C, 80.76; H, 9.15; N, 4.71; found: C, 81.15; H, 9.42; N, 4.76.

**3´-Hydroxy-3´-phenyl-2´-(1,1,3,3-tetramethylindan-2-ylidene)propionitrile (8).** A solution of LDA (1.14 mmol) in dry Et_2_O was prepared according to the GP and treated with the acrylonitrile derivative **1** (200 mg, 0.95 mmol). The yellow solution (**2Li**, 0.95 mmol) was decolorized on the addition of benzaldehyde (**5**, 0.126 mL, 1.24 mmol). Warm-up (GP) afforded a colorless powder (262 mg, 87%); this almost pure material was recrystallized from toluene (5 mL) to furnish a powder (108 mg, 36%) with mp 190–192 °C; **8** was insoluble in CCl_4_. ^1^H NMR (CDCl_3_, 400 MHz) δ 1.58 (s, 3H, 1 × 3-CH_3_), 1.71 and 1.72 (2 s, 2 × 3H, 2 × 1-CH_3_), 1.75 (s, 3H, 1 × 3-CH_3_), 2.33 (d, ^3^*J* = 6.7 Hz, 1H, OH), 6.15 (d, ^3^*J* = 6.7 Hz, 1H, H–CO), 7.17 and 7.20 (2 m, 2 × 1H, 4-/7-H), 7.30 (m, 2H, 5-/6-H), 7.35 (t, ^3^*J* = 7.0 Hz, 1H, *para*-H), 7.42 (tm, ^3^*J* = 7.2 Hz, 2H, 2 × *meta*-H), 7.51 (dm, ^3^*J* = 7.4 Hz, 2H, 2 × *ortho*-H) ppm, assigned through comparison with **7**; ^13^C NMR (CDCl_3_, 100.6 MHz) δ 28.8 and 29.0 (2 q, 2 × 1-CH_3_), 30.6 and 31.5 (2 q, 2 × 3-CH_3_), 49.3 (quat, C-3), 49.5 (quat, C-1), 69.6 (d, HCO), 112.4 (quat, C-α), 116.9 (quat, C≡N), 122.1 (d, C-4), 122.5 (d, C-7), 126.1 (d, 2 × C-*ortho*), 127.9 and 128.0 (2 d, C-5/-6), 128.4 (d, C-*para*), 128.8 (d, 2 × C-*meta*), 140.0 (quat, C-*ipso*), 147.6 (quat, C-7a), 147.8 (quat, C-3a), 179.5 (quat, C-2) ppm, assigned through comparison with **7** and benzyl alcohol; IR (KBr): 3404 (O–H), 2971, 2228 (C≡N), 1487, 1458, 1368, 1052, 751, 716 cm^−1^; anal. calcd for C_22_H_23_NO (317.4): C, 83.24; H, 7.30; N, 4.41; found: C, 83.39; H, 7.33; N, 4.48.

**3´-Cyclopropyl-3´-hydroxy-2´-(1,1,3,3-tetramethylindan-2-ylidene)propionitrile (9).** Using the GP protocol, the acrylonitrile derivative **1** (200 mg, 0.95 mmol) was deprotonated with LDA (1.05 mmol) in Et_2_O to generate **2Li** which was treated with cyclopropanecarboxaldehyde (**6**, 0.092 mL, 1.24 mmol). The crude product (259 mg) was crystallized from ethanol (2 mL) to give colorless **9** (126 mg, 47%) with mp 146–149 °C. ^1^H NMR (CDCl_3_, 400 MHz) δ 0.39 (m, 1H of cyclopropyl-CH_2_ *cis* to HCO), 0.65 (m, 2 × *trans*-H), 0.76 (m, 1 *cis*-H), 1.41 (m, 1H, *tert*-CH of cyclopropyl), 1.49 and 1.57 (2 s, 2 × 3H, 2 × 3-CH_3_), 1.69 and 1.71 (2 s, 2 × 3H, 2 × 1-CH_3_), 1.98 (d, ^3^*J* = 4.6 Hz, 1H, exchangeable with D_2_O, OH), 4.35 (dd, ^3^*J* = 7.7 and 4.6 Hz, 1H, H–CO), 7.14 and 7.18 (2 m, 2 × 1H, 4-/7-H), 7.27 (m, 2H, 5-/6-H) ppm, assigned through comparison with **7**; ^13^C NMR (CDCl_3_, 100.6 MHz) δ 2.8 and 3.8 (2 t, 2 diastereotopic cylopropyl-CH_2_), 16.4 (d, *tert*-C of cyclopropyl), 28.83 and 28.88 (2 q, 2 × 1-CH_3_), 30.3 and 32.0 (2 q, 2 × 3-CH_3_), 49.11 and 49.16 (2 quat, C-3 and C-1), 71.2 (d, HCO), 112.3 (quat, C-α), 117.4 (quat, C≡N), 122.1 (d, C-4), 122.5 (d, C-7), 127.76 and 127.87 (2 d, C-5/-6), 147.71 (quat, C-7a), 147.92 (quat, C-3a), 178.6 (quat, C-2) ppm, assigned as above; IR (KBr): 3471 (O–H), 2993, 2961, 2928, 2214 (s, C≡N), 1629, 1487, 1461, 1039, 759 cm^−1^; anal. calcd for C_19_H_23_NO (281.4): C, 81.10; H, 8.24; N, 4.98; found: C, 80.92; H, 8.11; N, 4.97.

**α-(2´-Hydroxyadamantan-2´-yl)-α-(1,1,3,3-tetramethylindan-2-ylidene)acetonitrile (13).** The technique of the GP was used to generate **2Li** in Et_2_O from LDA (1.05 mmol) and acrylonitrile derivative **1** (200 mg, 0.95 mmol). After continued stirring till **1** was completely dissolved, a solution of adamantan-2-one (**11**, 186 mg, 1.24 mmol) in dry Et_2_O (4 mL) was added slowly (1 drop per s) at rt, whereupon the mixture was stirred for 30 min and then worked up (GP). The crude material (321 mg after drying in presence of solid KOH) was recrystallized from CCl_4_ (5 mL) to give colorless platelets **13** (141 mg, 41%) with mp 179–180.5 °C. ^1^H NMR (CDCl_3_, 400 MHz, 25 °C) δ 1.64 (s, 6H, 2 × 3-CH_3_), 1.68 (dm. ^2^*J* = 12.5 Hz, 2H, 1 × 4´-H and 1 × 9´-H), 1.74 (broadened t, ^3^*J* ≈ 2 Hz, 2H, 2 × enantiotopic 6´-H), 1.79 (s, 6H, 2 × 1-CH_3_), 1.83 (m, ^3^*J* ≈ 2.5 Hz, 1H, 5´-H), 1.85 (dm, ^2^*J* obscured, 2H, 1 × 8´-H and 1 × 10´-H), 1.86 (s, 1H, OH, δ = 2.30 ppm at –60 °C), 1.89 (m, ^3^*J* = 2.8 Hz, 1H, 7´-H), 1.96 (broadened d, ^2^*J* = 13 Hz, 2H, 1 × 8´-H and 1 × 10´-H), 2.33 (broadened d, ^2^*J* = 12.5 Hz, 2H, 1 × 4´-H and 1 × 9´-H), 2.70 (broad, 2H, 1´-/3´-H), 7.12 (m, 1H, 4-H), 7.17 (m, 1H, 7-H), 7.26 (m, 2H, 6-/5-H) ppm, assigned through the NOESY correlations 4-H ↔ 3-CH_3_ ↔ 1´-/3´-H ↔ all four 4´-/9´-H ↔ 5´-H ↔ 2 × 6´-H ↔ 7´-H ↔ 8´-/10´-H ↔ 1´-/3´-H ↔ OH, and 7-H ↔ 1-CH_3_ (this without any cross-peaks to the adamantane protons); ^13^C NMR (CDCl_3_, 100.6 MHz) δ 25.9 (d, CH-5´), 26.5 (d, CH-7´), 28.8 (q, 2 × 1-CH_3_), 30.0 (q, 2 × 3-CH_3_), 32.9 (t, CH_2_-4´-/9´), 35.6 (t, CH_2_-8´-/10´), 37.0 (t, CH_2_-6´), 37.4 (d, CH-1´-/3´), 50.5 (quat, C-3), 51.5 (quat, C-1), 77.1 (quat, C-2´), 118.2 and 119.4 (2 × quat, C-α or C≡N), 121.9 (d, C-4), 122.5 (d, C-7), 127.45 and 127.57 (2 d, C-5/-6), 147.4 (quat, C-7a), 149.2 (quat, C-3a), 182.6 (quat, C-2) ppm, assigned through DEPT, HSQC, and comparison with **7**–**9**, no other significant changes at −60 °C; IR (KBr): 3471 (sharp, O–H), 2855, 2201 (sharp, C≡N), 1602 (w), 1488, 1453, 1353, 1020, 760 (s) cm^−1^; anal. calcd for C_25_H_31_NO (361.53): C, 83.06; H, 8.64; N, 3.87; found: C, 82.90; H, 8.50; N, 3.61.

**α-(9´-Hydroxyfluoren-9´-yl)-α-(1,1,3,3-tetramethylindan-2-ylidene)acetonitrile (18).** Using the GP protocol, the solid acrylonitrile derivative **1** (200 mg, 0.95 mmol) was added to a solution of LDA (1.05 mmol) in dry Et_2_O, followed by fluoren-9-one (**15**, 223 mg, 1.24 mmol) which changed the color of the mixture from yellow to dark green. The crude material (406 mg) obtained after work-up (GP) was recrystallized from toluene (9 mL) to afford colorless **18** (147 mg, 40%) with mp 243–245 °C. ^1^H NMR (CDCl_3_, 400 MHz, 25 °C) δ 1.66 (sharp s, 6H, 2 × 1-CH_3_), 1.98 (broadened s, 6H, 2 × 3-CH_3_), 2.35 (s, 1H, OH), 7.15 (dm, 1H, 7-H), 7.22 (broadened d, 1H, 4-H, narrowed at −45 °C), 7.25 (td, 1H, 5-H), 7.28 (td, 1H, 6-H), 7.33 (td, ^3^*J* = 7.5 Hz, 2H, 2´-/7´-H), 7.42 (td, ^3^*J* = 7.0 Hz, 2H, 3´-/6´-H), 7.48 (d, ^3^*J* = 7.6 Hz, 2H, 1´-/8´-H), 7.69 (d, ^3^*J* = 7.5 Hz, 2H, 4´-/5´-H) ppm, assigned through comparison with **13** and the NOESY correlations 3-CH_3_ ↔ 1´-/8´-H ↔ 1-CH_3_ (weaker) ↔ 7-H ↔ 6-H, and 1´-/8´-H ↔ 2´-/7´-H ↔ 3´-/6´-H ↔ 4´-/5´-H; ^1^H NMR (CDCl_3_, 400 MHz, −45 °C) δ 1.65, 1.98, 2.39, 7.20, 7.27, 7.30, 7.33, 7.37, 7.46, 7.49, 7.69 ppm; ^13^C NMR (CDCl_3_, 100.6 MHz) δ 29.1 (qq, ^1^*J* = 128 Hz, ^3^*J* = 4.5 Hz, 2 × 1-CH_3_), 31.7 (broadened qq, sharp at −45 °C, ^1^*J* = 128 Hz, ^3^*J* = ca. 4.5 Hz, 2 × 3-CH_3_), 50.1 (unresolved m, C-3), 51.0 (unresolved m, C-1), 85.7 (m, C-9´), 111.1 (very weak d, stronger at −45 °C, ^3^*J* = 8.5 Hz to OH, C-α), 117.9 (sharp s, C≡N), 120.6 (dd, ^1^*J* = 160 Hz, ^3^*J* = 8 Hz, CH-4´/-5´), 122.3 and 122.4 (2 dd, ^1^*J* = 159 Hz, ^3^*J* = 8 Hz, CH-7/-4), 124.1 (dd, ^1^*J* = 160 Hz, ^3^*J* = 8 Hz, CH-1´/-8´), 127.2 and 127.5 (2 dd, ^1^*J* = 160 Hz, ^3^*J* = 7.5 Hz, CH-5/-6), 128.8 (dd, ^1^*J* = 162 Hz, ^3^*J* = 7.3 Hz, CH-2´/-7´), 130.1 (dd, ^1^*J* = 159 Hz, ^3^*J* = 7.2 Hz, CH-3´/-6´), 139.9 (broadened t, ^3^*J* = 7 Hz, C-4a´/4b´), 147.4 (m, C-7a), 147.9 (t, ^3^*J* = 7.2 Hz, C-8a´/9a´), 149.6 (unresolved m, C-3a), 179.4 (m, C-2) ppm, assigned through comparison with **7**, **8**, **9**, and fluoren-9-one → HETCOR → COLOCS(7 Hz); COLOCS cross-peaks of ^3^*J*: 1-C*H*_3_ → C-2 and C-7a, 3-C*H*_3_ → C-2 and C-3a, OH → C-α, 4-H → C-6, 5-H → C-7, 6-H → C-4, 7-H → C-5, 1´-H → C-3´ and C-4a´, 2´-H → C-4´ and C-9a´, 3´-H → C-1´ and C-4a´, 4´-H → C-2´ and C-9a´; IR (KBr): 3525 (w). 3417 (s), 3022, 2959, 2925, 2215 (sharp, C≡N), 1607, 1488, 1451, 1366, 1207, 771, 761, 754, 735 cm^−1^; anal. calcd for C_28_H_25_NO (391.5): C, 85.90; H, 6.44; N, 3.58; found: C, 86.13; H, 6.04; N, 3.60.

**Cyclopropyl 1-[α,α-(dicyclopropyl)hydroxymethyl]cyclopropyl ketone (21)** [9]. ^1^H NMR (CDCl_3_, 400 MHz) δ 0.27 (m, 4H), 0.49 (m, 4H), 0.80 (m, 2H), 0.86 (m, 4H), 0.98 (m, 2H), 1.27 (m, 2H), 1.46 (m, 1 *tert*-H), 4.3 (broadened s, 1H, OH) ppm; ^13^C NMR (CDCl_3_, 100.6 MHz) δ 0.28 (2 × CH_2_), 0.39 (2 × CH_2_), 11.1 (2 × CH_2_), 11.3 (2 × CH_2_), 15.7 (1 × CH), 17.6 (2 × CH), 39.8 (1 × quat C), 70.7 (1 × quat, C–O), 212.9 (1 × quat, C=O) ppm.

## Supporting Information

File 1 Ion-pair intermediate through desilylation with Bu_4_N^+^ F^−^.
