# Supplementary material for: Nucleofugal behavior of a β-shielded α-cyanovinyl carbanion
Source: Beilstein J Org Chem. 2018 Dec 11;14:3018–24. doi: 10.3762/bjoc.14.281 (PMC6296431; doi:10.3762/bjoc.14.281)
Supplement: File 1 — Ion-pair intermediate through desilylation with Bu4N+ F−. [file Beilstein_J_Org_Chem-14-3018-s001.pdf]

BEILSTEIN JOURNAL OF ORGANIC CHEMISTRY

Supporting Information

for

Nucleofugal behavior of a β-shielded α-cyanovinyl carbanion

Rudolf Knorr and Barbara Schmidt

Beilstein J. Org. Chem. **2018,** *14,* 3018–3024. doi:10.3762/bjoc.14.281

Ion-pair intermediate through desilylation with $Bu_4N^+ F^-$

License and Terms: This is a supporting information file under the terms of the Creative Commons Attribution License (http://creativecommons.org/licenses/by/4.0). Please note that the reuse, redistribution and reproduction in particular requires that the authors and source are credited.
The license is subject to the *Beilstein Journal of Organic Chemistry* terms and conditions: (https://www.beilstein-journals.org/bjoc)

The metal-free ion-pair intermediate S3 is generated through desilylation.

Some of the following formula numbers (Scheme S1) are accompanied in parentheses by the corresponding numbers from the Main Text.

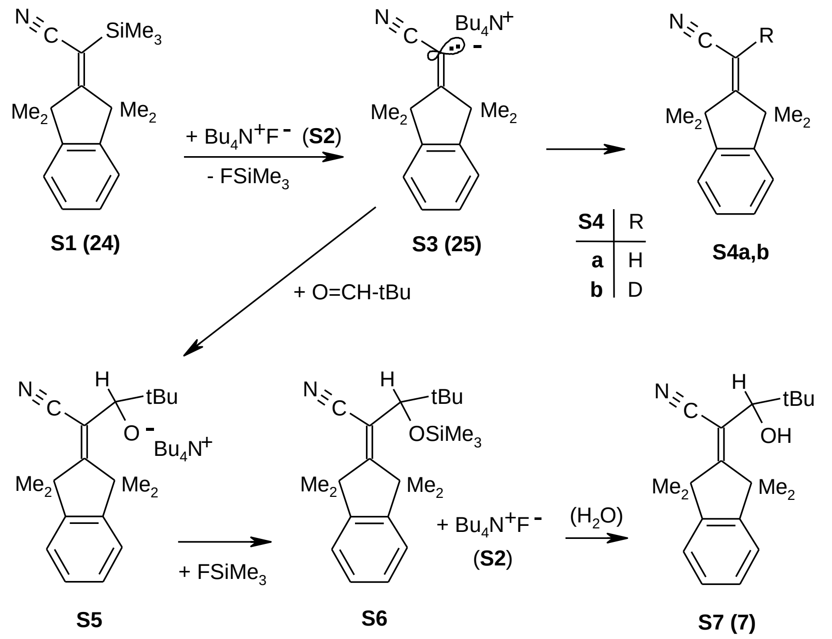

Scheme S1: Desilylation of **S1** with production of alkenes **S4a,b** or aldehyde adduct **S7**.

The known [S1] α-(1,1,3,3-tetramethylindan-2-ylidene)-α-trimethylsilylacetonitrile (**S1** (**24**)) can be desilylated (Scheme S1) by soluble tetrabutylammonium fluoride (Bu_4N^+ F^- (**S2**)): Formation of the metal-free ion pair **S3** in a $DCCl_3$ solution of **S1** and **S2** had been recognized [S1] through a deuterium transfer reaction that furnished the completely α-deuterated alkene **S4b** ([α-D]**1**). Trapping of **S3** with pivalaldehyde (O=CH-*t*-Bu) was now possible in THF as the solvent at room temperature with catalytic amounts of **S2** (≤0.05 equiv) which consumed **S1** almost immediately and yielded roughly equal amounts of alkene **4a** (**1**) and the adduct **S7** (**7**) after aqueous work-up. The consumption of **S1** was slower with reduced amounts of catalyst **S2**, requiring typically 2 hours with 0.01 equiv of **S2** or ca. 21 hours with 0.0036 equiv, while the product ratio of **S4a** and **S7** was always roughly 1:1 and did not depend on the solvents (THF, Et_2O, *t*-BuOMe, or benzene) and on the period of time prior to work-up. This unexpected product stability was a consequence of the catalytic process which demanded that **S2** should be regenerated from the co-product $FSiMe_3$ with a concomitant trimethylsilyl transfer to the emerging reactive primary alkoxide **S5** to give **S6**. This known [S2] kind of $SiMe_3$ transfer protected the system from the expected nucleofugal escape of the carbanion unit until **S6** succumbed to desilylation by the

aqueous work-up procedure with hydrolysis to give **S7**. Thus, **S6** could not be characterized; but the system of Scheme S1 explained the surprising adduct stability and described the fate of the Bu_4N^+ intermediate in accord with a previously [S2] analyzed, different system.

The expected alkene **S4a** was observed already in situ (before work-up); therefore, its major portion cannot be ascribed to aqueous work-up or to the tiny water content of the completely dissolved reagent Bu_4N^+ F^- (0.004–0.05 equiv) but may perhaps result through HF elimination from Bu_4N^+ F^- and subsequent reactions. In any case, the formation of **S4a** from either unconsumed ion pair **S3** or cleaving Bu_4N^+ alkoxide **S5** remained an open question.

References

S1. Knorr, R.; Schmidt, B.; Mehlstäubl, J.; von Roman, T. *J. Organomet. Chem.* **2018**, *871*, 185–196.

S2. Briddle, M. M.; Reich, H. J. *J. Org. Chem.* **2006**, *71,* 4031–4039, Figure 1 and cited references therein.
